# Natural L-type calcium channels antagonists from Chinese medicine

**DOI:** 10.1186/s13020-024-00944-8

**Published:** 2024-05-21

**Authors:** Fangfang Xu, Wanna Cai, Bo Liu, Zhenwen Qiu, Xiaoqi Zhang

**Affiliations:** 1 The Second Clinical College , Guangzhou University of Chinese Medicine, Guangzhou, 510006 People’s Republic of China; 2 The First Affiliated Hospital, Guangzhou University of Chinese Medicine, Guangzhou, 510405 People’s Republic of China; 3 Guangdong Provincial Engineering Research Center for Modernization of TCM, NMPA Key Laboratory for Quality Evaluation of TCM, Jinan University, Guangzhou, 510632 People’s Republic of China

**Keywords:** LTCCs, Antagonists, Excitation–contraction coupling, TCM, Natural phytochemicals

## Abstract

L-type calcium channels (LTCCs), the largest subfamily of voltage-gated calcium channels (VGCCs), are the main channels for Ca^2+^ influx during extracellular excitation. LTCCs are widely present in excitable cells, especially cardiac and cardiovascular smooth muscle cells, and participate in various Ca^2+^-dependent processes. LTCCs have been considered as worthy drug target for cardiovascular, neurological and psychological diseases for decades. Natural products from Traditional Chinese medicine (TCM) have shown the potential as new drugs for the treatment of LTCCs related diseases. In this review, the basic structure, function of LTCCs, and the related human diseases caused by structural or functional abnormalities of LTCCs, and the natural LTCCs antagonist and their potential usages were summarized.

## Background

Voltage-gated calcium channels (VGCCs) are voltage-dependent heterogeneous transmembrane proteins located in cell membranes, which can be divided into high-voltage activated type and low-voltage gated type according to their conductivity and voltage sensitivity [1]. In mammals, *α*_1_ subunit, the core component of VGCCs, can be divided into three families with a total of 10 different channels, including Cav1 [L-type calcium channels (LTCCs), Cav1.1–1.4], Cav2 (Cav2.1–2.3) and Cav3 (T-type calcium channels, Cav3.1–3.3). The Cav2 family is consist of the P/Q-type calcium channels (Cav2.1), N-type calcium channels (Cav2.2), and R-type calcium channels (Cav2.3) [2].

LTCCs, the largest subfamily of VGCCs, are the main channel of Ca^2+^ influx in the cell excitatory process, which closely related to excitation–contraction coupling (ECC) and excitation-secretion coupling [3]. LTCCs are widely present in various excitable cells, especially cardiac and cardiovascular smooth muscle cells, which are essential for heart and nervous function [3]. Cav1.1 of LTCCs is distributed in skeletal muscle, and its mutation is associated with hypokalemic periodic paralysis type 1 (HPP-1) [4] and malignant hyperthermia [5]. Cav1.2 and Cav1.3 are primarily existed in the heart and brain, and are related to Timothy syndrome (TS) [6, 7], arrhythmia, bipolar disorder (BD) [8] and autism [7]. Cav1.4 is presented in the retina and variants of Cav1.4 lead to night blindness [9].

General speaking, regulation of LTCCs has been considered as an important strategy for treating diseases for decades. LTCCs antagonists have been used for the treatment of hypertension, arrhythmia and other diseases, which illustrated their therapeutic activities in myocardial ischemia protection (MI), myocardial and vascular wall hypertrophy prevention, atherosclerosis prevention, and renal protection [10, 11]. Clinical-used LTCCs antagonists can be divided as 1, 4-dihydropyridine, benzothiazole, and phenylalkyl amine according to their chemical structures [12].

Traditional Chinese medicine (TCM) has a long history in cardiovascular diseases, among which *Salvia miltiorrhiza*, *Ligusticum wallichii*, *Angelica sinensis*, *Paeonia lactiflora* and *Paeonia suffruticosa* exhibited the calcium antagonistic effect [13]. A total of 45 active ingredients from Chinese medicine with antihypertensive effect were screened though pharmacophore model based on drug repositioning method, which suggested that the Chinese medicine were the potential source of LTCCs antagonists [14]. Therefore, it is of great value to develop and design efficient LTCCs antagonists from TCM.

In this review, we summarized the basic structure and molecular functions of LTCCs, related diseases caused by channel dysfunction. In addition, the LTCCs antagonists with different types from natural products of TCM were also simiply summarized. Furthermore, we hope to discover new natural LTCCs antagonists with high specificity in treating human diseases.

### The structures and functions of LTCCs

**Fig. 1:**
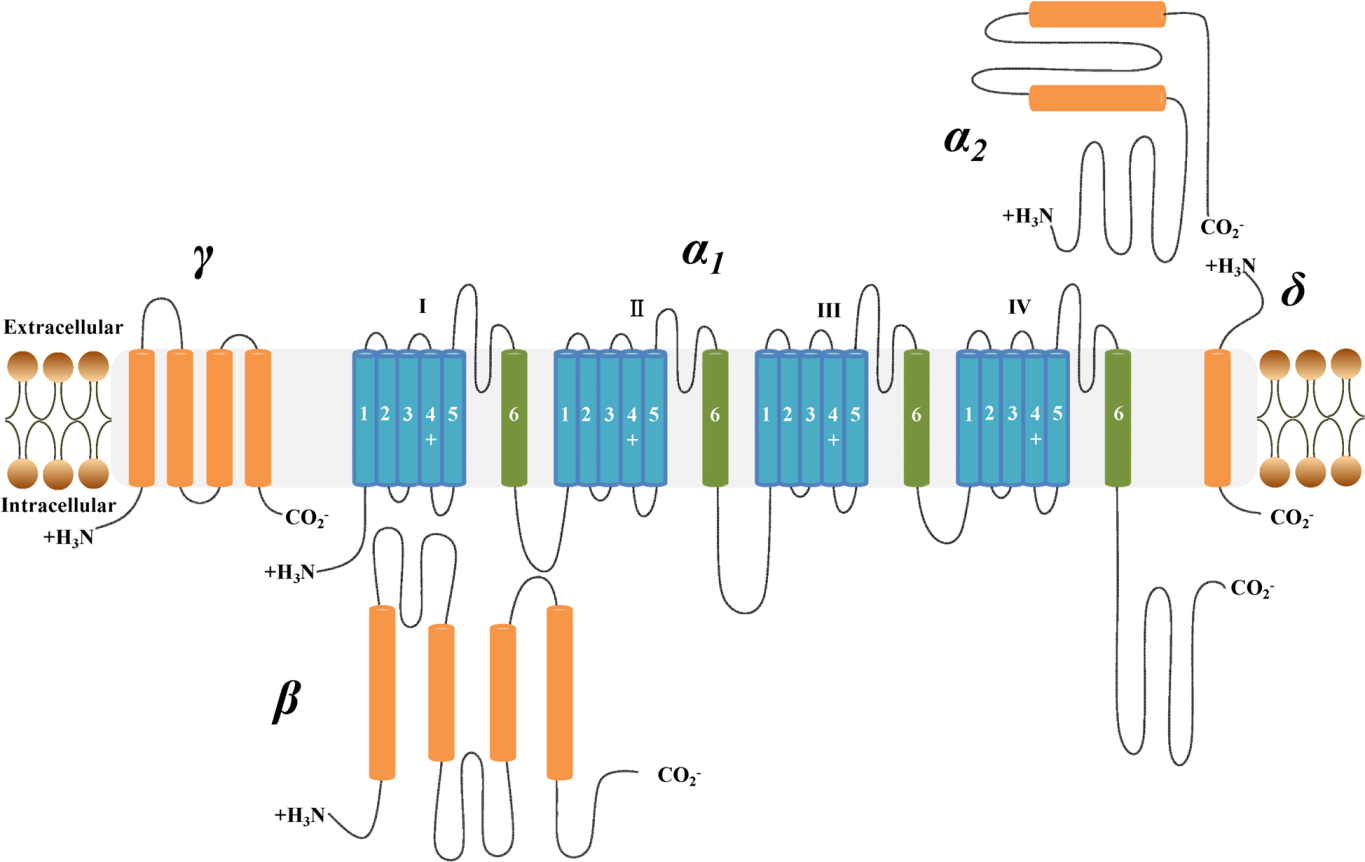
Subunit structure of LTCCs. It consists of *α*_1_, *α*_2_, *δ*, *β* and *γ* subunits, and the *α*_1_ subunit consists of four domains with six fragments in each domain (S1–S6). The positively charged S4 responds to the membrane potential change by transferring to the pore domain via the cytoplasmic S4–S5 connector. The motion of S4 is guided by the negatively charged interaction provided by the S1–S3

LTCCs is a polymeric transmembrane protein composed of *α*_1_, *α*_2_, *δ*, *β* and *γ* subunits [1]. The *α*_1_ subunits is the central part, which can be divided into four subtypes, and encoded by different genes, including *α*_1_S (Cav1.1), *α*_1_C (Cav1.2), *α*_1_D (Cav1.3) and *α*_1_F (Cav1.4) [2] (Table 1) (Fig. 1).

**Table 1:** Classification and pharmacology of LTCCs

Gene	*α*_1_Subunits (old nomenclature)	Predominant tissue expression	Principal physiological functions	Related diseases
CACNA1S	Cav1.1 (*α*_1_S)	Skeletal muscle	EC coupling in skeletal muscle, regulation of transcription	HPP-1, malignant hyperthermia susceptibility
CACNA1C	Cav1.2 (*α*_1_C)	Cardiovascular, endocrine and nervous system	EC coupling and excitation-transcription coupling in cardiac and smooth muscle, endocrine secretion, neuronal Ca^2+^ transients	MI/RI, TS, PD, AD, febrile seizures and TSC
CACNA1D	Cav1.3 (*α*_1_D)	Nervous, endocrine, cardiovascular system; cochlea cells	Neuronal Ca^2+^ transients, cardiac pacemaking, endocrine secretion, auditory transduction	BrS, PD, AD, BD, schizophrenia, APAs and CHI
CACNA1F	Cav1.4 (*α*_1_F)	Retina, mast cells	Visual transduction	CSNB2

Cav1.1-Cav1.4 distributes in different tissues or organs (Table 1). The Cav1.1, known as dihydropyridine receptor, needs to work along with type 1 ryanodine receptor (RyR1), that is mainly distributed in skeletal muscle [15]. Cav1.2 and Cav1.3 are mainly located in adrenal cardiac, neuronal and chromaffin cells. Cav1.3 is more sensitive than Cav1.2, and Cav1.3 can be triggered at low voltages and inactivated at the voltage-dependent manner. Cav1.4 is largely localized in the retinal cells and affects the release of neurotransmitter and Cav1.4 influences the dihydropyridine-sensitivity without the Ca^2+^ currents [9].

The auxiliary subunits *β* belongs to the MAGUK-scaffolding protein family, a cytosolic soluble protein with high affinity binding to channel, including four subtypes of *β*_1_-*β*_4_ [16]. The mutation of *β* subunit is associated with arrhythmia and stroke [16]. The α2*δ* subunits are encoded by one unique gene, and translationed into two separate proteins that linked by disulfide bond. The four subtypes of α2*δ* proteins, α2*δ*_*1*_-α2*δ*_*4*_, function as a thrombospondin receptor to regulate excitatory synpatogenesis [2, 16]. There are eight subtypes of *γ* subunit (*γ*_1_-*γ*_8_), that is composed of four transmembrane helical segments with intracellular amino (NH2) and carboxy (COOH) termini. The physiological function of the *γ* subunit needs further research to reveal [2, 16]. The complex structure of Cav1.1 (*α*1, *α2δ*, *β*, and *γ*) from rabbit skeletal muscle membranes were determined by cryo-EM, which layed foundation for further understanding the working mechanisms of LTCCs with important physiological and pathological functions [17, 18].

### LTCCs dysregulation—associated human diseases

#### Cardiovascular disease

Ca^2+^ is involved in many cellular processes such as EC coupling, membrane excitability and transcriptional regulation of cardiomyocytes through LTCCs influx, and plays an important role in physiological functions of cardiomyocytes [11]. The Cav1.2, Cav1.3 and auxiliary subunits, including *β* subunits, *α*_2_*δ* subunits in myocardium, participate in the regulation of the activation and inactivation characteristics of the channels [11, 19]. The disorder of LTCCs can lead to electrophysiological abnormalities, arrhythmias, and various Ca^2+^ dependent dysfunctions in cellular processes.

Myocardial ischemia/reperfusion injury (MI/RI) refers to the severe injury of ischemic myocardial tissue after restoration of perfusion and the clinical manifestations include reperfusion arrhythmia, intracardial hemorrhage and myocardial infarction [20]. Calcium homeostasis is essential for maintaining ECC in cardiomyocytes, including calcium release, recapture, and storage [10]. Studies have shown that MI/R injury can cause disruption of calcium homeostasis and Ca^2+^ overload is one influencing factors in MI/RI, which may lead to energy metabolism disorder, myocardial hypoxia and ultimately myocardial cell necrosis [21]. The regulatory proteins, including LTCC, provide potential targets for the prevention and treatment of clinical MI/RI.

Hypertension is closely associated with increased vascular contraction. The influx of Ca^2+^ into the vascular smooth muscle cells produce membrane potential and increase the tension of the blood vessel, which affect the arterial contraction and blood pressure [22]. Moreover, the calcium sensitization process in smooth muscle cells could increase the vascular smooth muscle contraction though DAG-PLC-PKC pathway and the RhoA-Rho kinase pathway [23].

The mutation of the LTCCs causes the imbalance of Ca^2+^ in cells and the changes of membrane potential, which causing the myocardial cells to be unusually excited and eventually lead to cardiac dysfunction [19]. The mutations of *α*_1_C and *β*_2b_ may lead to Idiopathic ventricular fibrillation. The absence of CACNA1C p.E850 may reduce the surface expression of LTCC, which leading to a significant reduction of I_Ca_. Inactivation of Cav1.3 bring out a strong decrease of I_Ca_ in the sinoatrial nodal pacemaker cells, which resulting in sinoatrial node dysfunction manifested as sinoatrial arrhythmia and bradycardia [24]. Brugada syndrome (BrS) is an inherited arrhythmia related to mutations of 18 different genes, of which *α*_1_C mutation ranked the second common cause. In particular, CACNA1C and CACNB2 mutations accounted for 12% of BrS cases. The latest study identified two BrS-related mutation sites between domains I and II of Cav1.2, including p.T320M and p.Q428E [25].

#### Neurological disorders

LTCCs are also essential for neuronal functions. The mutations in LTCCs genes have a close relationship with various neurological and psychiatric disorders, including Timothy syndrome (TS), Parkinson’s disease (PD), Alzheimer’s disease (AD), epilepsy, Tuberous sclerosis complex (TSC) and drug addiction.

Timothy syndrome (TS) is a debilitating and multiorgan disease involving mental retardation, fatal arrhythmias and autism [6, 7]. Studies revealed that Cav1.2 channel mutations in TS patients leaded to impaired neural circuits [26]. In addition, genome-wide association studies have found significant associations between *α*_1_C intron SNPs and psychiatric disorders including BD, schizophrenia and autism spectrum disorders [6]. The characteristics of typical and atypical TS phenotypes have been summarized in a recent review to elucidate the molecular mechanism of Cav1.2 gated dysfunction leading to mental illness [27].

Dysregulation of calcium homeostasis is a compensatory result of neurodegenerative processes in early Parkinson’s disease (PD). The amount of Cav1 subtypes and the calcium-binding proteins in PD were different from control group. The increased expression of Cav1.3 subtype in the cerebral cortex of early stage PD patients may subjoin the cells metabolic burden that depend on LTCCs subtypes for electrical activity, which lead to the neurodegeneration of specific groups of neurons. The change in ratio of Cav1.2 to Cav1.3 in parkinsonian brain could increase neuronssusceptible to excitotoxicity or oxidative stress [28].

Alzheimer’s disease (AD) is characterized by the accumulation of *β*-amyloid peptide (A*β*), dysfunction of synapses, and loss of neurons. The increasement of age associated oxidative stress and metabolic disorders cause dysplasia of calcium homeostasis. The extracellular accumulation of A*β* enhance calcium load and increase the current of the Cav1.2 and Cav1.3 in AD [29]. Moreover, blocking calcium channels alleviate amyloid-*β*-induced neuronal decline in vitro and exhibited neuroprotective effects [30].

Epilepsy refers to the recurrent brain dysfunction resulted from sudden excessive and disordered neuronal discharge. Nimodipine can block abnormal spontaneous activity of hippocampal pyramidal neurons of heat-induced in Cav1.2 knock-out mice brain slices, suggesting that Cav1.2 subunit is critical in temperature-dependent intrinsic firing of febrile epilepsy [31].

Tuberous sclerosis complex (TSC) is neurologic impairment that associated with epilepsy. The development of TSC epilepsy closely related with high activity of *TSC2*-deficient (*TSC2*^−/−^) neurons. The specific inhibitor of mTOR protein, rapamycin, could inhibit the abnormal increase of LTCCs subtype Cav1.3 in *TSC2*^−/−^ neurons. The results indicated that LTCCs were critical downstream component of TSC-mTOR signal and can trigger the enhancement of network activity of *TSC2*^−/−^ neurons [32]. Therefore, LTCCs may be a new therapeutic target for TSC epilepsy.

Drug addiction, known as drug dependence or drug abuse, is a stubborn and chronic recurrent neurological disease. Ca^2+^ ions and Ca^2+^ channels are involved in the formation of drug addiction, and L-type Ca^2+^ channels are an important target for anti addiction drug research [33, 34]. Studies showed that Cav1.2 channels, rather than Cav1.3, are involved in withdrawal reaction in nicotine-induced abuse and alcohol-seeking abuse [35, 36]. LTCC blockers, such as dihydropyridines, have been considered a potential therapeutic drug to alleviate the symptoms of drug addiction [37].

#### Psychological diseases

Several studies have implicated that LTCCs disorders may lead to psychiatric ills, such as BD, and schizophrenia [7, 9, 19], which suggested the importance of LTCCs in learning, memory, and synaptic plasticity.

The Genomic data suggested that *CACNA1S, CACNA1C* and *CACNA1D* were the core genes that related with psychiatric diseases. Calcium signaling dysfunction is one pathogenic factor for psychological diseases [38–41]. The subunit *α*_1_C, *α*_1_B and *β*_2_ subunits were risk locus for BD, schizophrenia and recurrent major depression [38, 42]. The microRNA 137 has been proved as a potentially risk for schizophrenia, and the *CACNA1C*, one target of microRNA 137, influenced the development process of schizophrenia [39].

LTCCs antagonists has been used for the treatment and prophylaxis of psychological diseases over 30 years, but has not turned into an established therapeutic scheme [8]. The results of existing studies about LTCCs antagonists on other phases of the illness were limited to the observational research without robust evidence. However, long-term or excessive usage of LTCC antagonists increased the risk of cardiovascular disease and depression [40]. Thus, it is important to comprehensive utilize the pharmacological, molecular, and genetic material to ameliorate the efficacy, safety and selectivity of LTCCs antagonists in the clinical treatment of psychological diseases.

#### Other diseases

##### Hypokalemic periodic paralysis type 1 (HPP-1)

Familial HPP-1 is an autosomal dominant disorder caused by *CACNA1S* encoding LTCCs Cav1.1, which resulting in abnormal voltage sensing and affecting skeletal muscle function [4]. Recently, Cav1.1 channel channelopathies in skeletal muscle have been reviewed [5]. Many muscle diseases have been identified to be associated with *CACNA1S* mutations, including hypo- and normokalemic periodic paralysis, malignant hyperthermia susceptibility, Cav1.1-related myopathies, and myotonic dystrophy type 1 [5].

##### Aldosterone-producing adenomas (APAs)

APAs account for approximately 50% of primary aldosteronism, which is one of the most common causes for hypertension patients. Zona glomerulosa (ZG)-like APAs have four somatic mutations, V259D, G403R, I750M and P1336R, in the Ca^2+^ pore at the S5 and S6 domains of Cav1.3 [43]. Selective Cav1.3 blockers may treat ZG-like APAs hyperaldosteronism without the vascular side effects caused by current LTCCs blockers.

##### Congenital hearing impairment

Congenital hearing impairment has extensive genetic heterogeneity. Cav1.3 is expressed in cochlear hair cells and is critical for auditory brainstem development [9]. *α*_1_D mutations in Cav1.3 was found in two consanguineous families with deafness and severe mouse sinoatrial node dysfunction with bradycardia [44].

##### Congenital stationary night blindness type 2 (CSNB2)

CSNB2 patients exhibit some degrees of night blindness, low visual acuity and myopia [45]. Cav1.4 of LTCCs is mainly expressed in retinal neurons, especially at the photoreceptor terminals. The mutations in the *CACNA1F* gene that encodes Cav1.4 channels lead to the the channel activity altered and caused the retinal disease, for example, CSNB2 [9]. The different structural, functional phenotypes and treatment options of Cav1.4 mutations in CSNB2 were summarized in recent review [46]. The gene therapeutic maybe a promising approach to CSNB2 patients in future.

### LTCCs antagonists from natural products

**Fig. 2:**
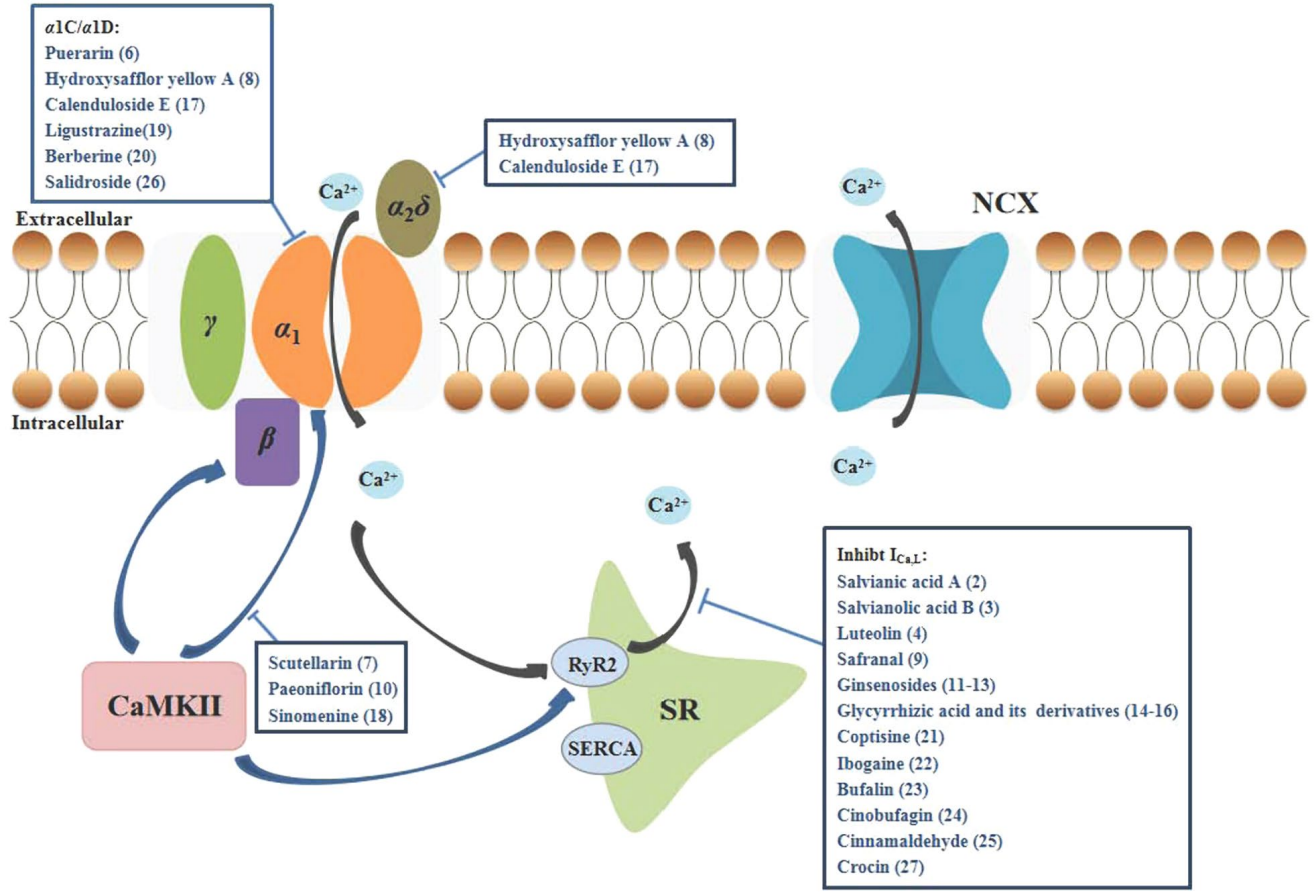
The LTCCs antagonists from natural products. The excitation–contraction coupling process begins with the entry of Ca^2+^ into the cell through LTCCs, followed by the triggering of Ca^2+^ release on SR by RyR2, and ultimately triggering intracellular Ca^2+^ concentration mediated contraction. Subsequently, Ca^2+^ in the cytoplasm is brought back to SR by SERCA and transported back to extracellular space via NCX (black arrow). Activated CaMKII induces stimulatory actions by phosphorylating major Ca^2+^ homeostatic proteins, activating I_Ca,L_ (mediated by the Thr498 terminal of *α* and *β*_2a_ subunits), phospholamban to increase cytosolic Ca^2+^ uptake by the SR, and RyR to increase SR Ca^2+^ release (blue arrow). Some active ingredients in TCM can antagonize LTCCs through various mechanisms. Inhibition of LTCCs subunits (*α*_1_C, *α*_1_D, *α*_2_*δ*), CaMKII phosphorylation and I_Ca,L_ reduced the release of Ca^2+^ from the sarcoplasmic reticulum

LTCCs are implicated in multiple cardiovascular, neurological and psychological diseases, and has become an important target for drug development. Natural products have been considered as valueable sources for drug discovery as their fewer adverse effects and multiple mechanisms. Many TCM active ingredients have been reported with inhibitory effect on LTCCs through various mechanisms (Table 2), including reducing the expression of Cav1.2 and Cav1.3 subunits and related proteins, inhibiting calcium channel currents, restricting calcium influx, and decreasing calmodulin-dependent protein kinase II (CaMKII) signaling pathways (Fig. 2).

**Table 2:** Pharmacological effect and mechanism of natural products of TCM on LTCCs

Compound	Disease	Model	Mechanism involved in inhibition of LTCCs	Effects	Refs.
Paeonol (**1**)	Myocardial infarction, MI and other cardiovascular diseases	The superior mesenteric artery removed from Sprague–Dawley (SD) rats were precontracted with 60 mmol/L KCl	Inhibit VDCC-mediated extracellular Ca^2+^ influx and receptor-mediated Ca^2+^ influx and release	Non-endothelium-dependent-vasodilatation in rat mesenteric artery	[48]
	Primary dysmenorrhea	Female ICR mice were administered Oxytocin (100*μ*/kg) to induce dysmenorrhea	Activate CB2R inhibits LTCCs extracellular Ca^2+^ influx through MAPK/ERK pathway	1. Ameliorate dysmenorrhea and uterine inflammation in mice 2. Restrain calcium influx and uterine contractions in a CB2R-dependent manner	[50]
Salvianic acid A (**2**)	MI, IHD	Isoproterenol (ISO)-induced MI in SD rats	1. Inhibit I_Ca,L_ 2. Decrease the release of sarcoplasmic reticular Ca^2+^	Dose-dependently reduce I_Ca,L_ and contractility in rat ventricular myocytes via decreasing the myocardial oxygen consumption	[52]
Salvianolic acid B (**3**)	MI	Ventricle myocytes of SD rats	Inhibit I_Ca,L_	Inhibit I_Ca,L_ and myocardial contractility without causing drug-induced LQTS	[53]
	Hypertension	Isolated coronary artery rings of SD rats precontracted with 5-hydroxytryptamine	1. The inhibition of Ca^2+^ influx in the vascular smooth muscle cells 2. The opening of potassium (K^+^) channels	Non-endothelium-dependent-vasodilatation in rat coronary artery	[54]
Luteolin (**4**)	MI/RI	Myocardial ischemia reperfusion model of SD rats	Inhibit I_Ca,L_	1. Protect heart structure 2. Reduce myocardial cell apoptosis 3. Prevent Ca^2+^ overload and increas vessel dilation	[55, 56]
Calycosin (**5**)	Cardiovascular diseases	Vasoconstriction of SD rats induced by KCl or PHE	Decrease extracellular Ca^2+^ influx through VOC and ROC	Inhibit vasoconstriction induced by KCl or PHE, and antagonize Ca^2+^-induced contraction in aortic rings	[57]
Puerarin (**6**)	Iron overload-induced injury	Iron-overloaded mice	1. Dose-dependently down-regulated Cav1.2 levels 2. Inhibit MAPK/ERK pathways	1. Regulate iron-handling proteins, decrease intracellular Fe^2+^, and inhibit cell apoptosis 2. Suppress the oxidative stress induced by iron overload	[59]
Scutellarin (**7**)	Cardiac hypertrophy	Cardiac hypertrophy of C57BL/6 mice induced by PHE	Inhibit Ca^2+^-mediated calcineurin and CaMKII pathways	1. Suppresse the hypertrophic growth of neonatal cardiac myocytes exposed to PHE 2. Inhibit heart subjected to pressure overload induced by aortic banding	[61]
Hydroxysafflor yellow A (**8**)	MI/RI and hypertension	Neonatal rat primary cardiomyocytes and human-induced pluripotent stem cell-derived cardiomyocytes (hiPSC-CMs)	1. Inhibit I_Ca,L_ 2. Reduce intracellular Ca^2+^ overload 3. Attenuate the higher expression of *α*1C and *α*_2_*δ*	1. Reduce the levels of myocardial enzymes 2. Restore the contractile function of hiPSC-CMs and exerted a protective effect on cardiac function 3. Decrease mitochondrial membrane potential and inhibit apoptosis and Ca^2+^ overload	[63]
Safranal (**9**)	IHD	ISO-induced MI in SD rats	1. Inhibit I_Ca,L_ and LTCC activity in the cardiomyocyte membrane 2. Regulate intracellular Ca^2+^ homeostasis	1. Reduce myocardial contractility and oxygen consumption 2. Inhibit oxidative stress 3. Inhibit LTCC and reduce Ca^2+^ overload	[64]
Paeoniflorin (**10**)	PMS, depression symptoms	PMS model of Wistar rats stimulated with leg binding	1. Inhibit I_Ca,L_ (Cav1.2) 2. Decrease the CaMKII protein level in the Cav1.2-induced CaM/CaMKII signalling pathway	1. Download CaM and p-CaMKII expression and increase the BDNF protein expression and reduce Ca^2+^ overload 2. Mitigate depressive behaviour	[65]
Ginsenoside Rb1 (**11**)	IHD	Myocardial cell ischemia model was established by 95%N_2_ + 5%CO_2_	Inhibit I_Ca,L_ by downing regulate the expression of Cav1.2	Shorten action potential duration of ischemic cardiomyocytes and inhibit the opening of LTCCs	[67]
		Myocardial ischemia reperfusion model of SD rats			[68]
Ginsenoside Re (**12**)	IHD	Myocardial cell ischemia model was established by by aconitine alkaloids	2. Reverse Cav1.2 mRNA level	Decrease injuries of the neonate rat cardiomyocytes	[70]
Ginsenoside Rd (**13**)	IHD	Myocardial ischemia reperfusion model of SD rats	1. Inhibit I_Ca,L_ 2. Active the Gi protein 3. The production of NO and the NO-cGMP signal pathway	1. Inhibit LTCCs and reduce Ca^2+^ overload 2. Reduce myocardial contractility and oxygen consumption	[69]
Glycyrrhizic acid (**14**)	Neurodegenerative disorder	MPP + induced damage to PC12 cells	Suppress intracellular Ca^2+^ overload	GA mitigated the calcium overload caused by MPP +	[72]
	IHD	ISO-induced MI in SD rats	1. Inhibit I_Ca,L_ 2. Reduce the Ca^2+^ transient	1. Inhibit LTCCs and reduce Ca^2+^ overload 2. Reduce the AMP of the ventricular myocardial cell contraction and oxygen consumption	[73]
Magnesium isoglycyrrhizinae (**15**)	IHD	Myocardial ischemia reperfusion model of SD rats	1. Inhibit ICa, L 2. Reduce the Ca2 + transient	1. Inhibit LTCCs and reduce Ca2 + overload 2. Reduce the AMP of the ventricular myocardial cell contraction and oxygen consumption 3. Have no influence on IKr	[75]
Monoammonium glycyrrhizinate (**16**)	IHD	ISO-induced MI in SD rats	1. Inhibit I_Ca,L_ 2. Reduce the Ca^2+^ transient	1. Inhibit LTCCs and reduce Ca^2+^ overload 2. Reduce myocardial contractility and oxygen consumption 3. Reduce the production of ROS, MDA, and SOD	[76]
Calenduloside E (**17**)	MI/RI	Myocardial ischemia reperfusion model of SD rats	1. Decrease the expression of *α*1C and *α*2*δ* 2. Enhance the interaction between LTCC and BAG3	1. Protect against MI/R injury 2. Recover damaged ARVMs and intracellular Ca^2+^ homoeostasis	[77]
Sinomenine (**18**)	Ischaemic brain injury	Neurons from neonatal SD rats, CHO cells, rat PC12 cells	1. Inhibit LTCCs and ASIC1a 2. Download the CaMKII phosphorylation level	1.The cytoprotection on PC12 cells 2. The neuroprotection on cerebral injury before ischemia or after injury 3.Reduce cerebral infarction	[79]
Ligustrazine/Tetramethylpyrazine (**19**)	IHD	Ventricular myocytes of adult New Zealand white rabbits	1. Inhibit I_Ca,L_ 2. Reduce intracellular Ca^2+^ overload	1. Inhibit LTCCs 2. Reduce myocardial contractility and oxygen consumption	[81]
	MSD	Soleus muscle of SD rats	1. Inhibit LTCC Cav1.3 2. Decrease the mRNA expression levels of caspase-3, caspase-9, Cav1. 3	1. Enhance the activity of Ca^2+^-ATPase and expression of RyR1 2. Inhibit expression of Cav1.3 3. Maintain the homeostasis of Ca^2+^ and inhibit the apoptosis of soleus muscle cells	[82]
	AD	Hippocampal neuronal cells of Wistar rats	1. Inhibit I_Ca,L_ 2. Reduce intracellular Ca^2+^ overload	1. Inhibit LTCCs 2. The cytoprotection on hippocampal neuronal cells	[83]
Berberine (**20**)	DM	Diabetic rats	1. Inhibit I_Ca,L_ 2. Reduce intracellular Ca^2+^ overload 3. Reduce the *α*_1_C-subunit expressions of LTCCs	1. Inhibit LTCCs 2. Reduce glucose levels 3. Inhibit cerebral artery contraction in diabetic rats	[86]
	AD	Streptozotocin-induced sporadic dementia of Alzheimer’s type in rats	Synergies with LTCCs blocker verapami	1. Improve cognitive performance and relieve neuroinflammatory 2. Attenuate oxidative stress in both hippocampus and frontal cortex 3. Attenuate the AChE activity and TNF-α level 4. Restore mitochondrial enzyme complex I, II, and IV activities	[87]
Coptisine (**21**)	Pulmonary diseases	Male BALB/c mice, mouse ASM cells	1. Inhibit VDLCC and NSCC currents 2. Reduce intracellular Ca^2+^ overload	1. Relax abnormal contracted mouse ASM 2. Block VDLCCs and NSCCs 3. Alter the intracellular Ca^2+^ concentration	[90]
Ibogaine (**22**)	Drug abuse	Adult ventricular guinea pig Cardiomyocytes, TSA-201 cells	Inhibit Cav 1.2 channel	1. Inhibit LTCC 2. Prolong the AP	[92]
Cinobufagin (**23**)	IHD	Adult SD rat ventricular myocytes	Inhibit I_Ca,L_	1. Inhibit LTCC 2. Alter the intracellular Ca^2+^ concentration	[93]
Bufalin (2**4**)	IHD	Adult rat ventricular myocytes	1. Inhibit I_Ca,L_ by reducing the Ca^2+^ current amplitude 2. Reduce intracellular Ca^2+^ overload	1. Inhibit LTCC 2. Alter the intracellular Ca^2+^ concentration 3. The negative inotropic action in myocardial cells	[94]
Cinnamaldehyde (**25**)	Hypertension	Male Wistar rats, male C57BL/6 mice, and blood pressure normal mice	1. In VCM and VSMC 2. Inhibit LTCC Cav1.2	1. Inhibit aortic contraction 2. Reduce Ca^2+^ concentration in VSMC and VCM	[95]
Salidroside (**26**)	Hypoxic-ischemic brain damage	Hippocampus neurons of SD neonatal rats	1. Inhibit LTCC Cav1.3 2. Decrease the mRNA expression levels of NMDAR1 and Cav1. 3 3.Reduce intracellular Ca^2+^ overload	1. Reduce cell damage caused by hypoxia of hippocampal neurons 2. Inhibit expression of Cav1.3 3. Maintain the homeostasis of Ca^2+^ and inhibit the apoptosis of soleus muscle cells	[96]
	Vascular complications of diabetis	Male diabetic Goto-Kakizaki and non-diabetic control Wistar-Kyoto rats	1. Inhibit I_Ca,L_ 2. Reduce the expressions of α_1_C-subunit at protein and mRNA levels in cerebral arteries	1. Lower blood glucose 2. Reduce blood pressure and alleviated cerebrovascular contractile activity 3. Inhibit the function and expression of Ca_L_ channel in cerebral VSMCs	[97]
Crocin (**27**)	IHD	Adult SD rat ventricular myocytes	1. Inhibit I_Ca,L_ and LTCCs activity in the cardiomyocyte membrane 2. Regulate intracellular Ca^2+^ homeostasis	1. Reduce myocardial contractility and oxygen consumption 2. Inhibit oxidative stress 3.Inhibit LTCCs and reduce Ca^2+^ overload	[99]

### Polyphenols

#### Paeonol

**Fig. 3:**
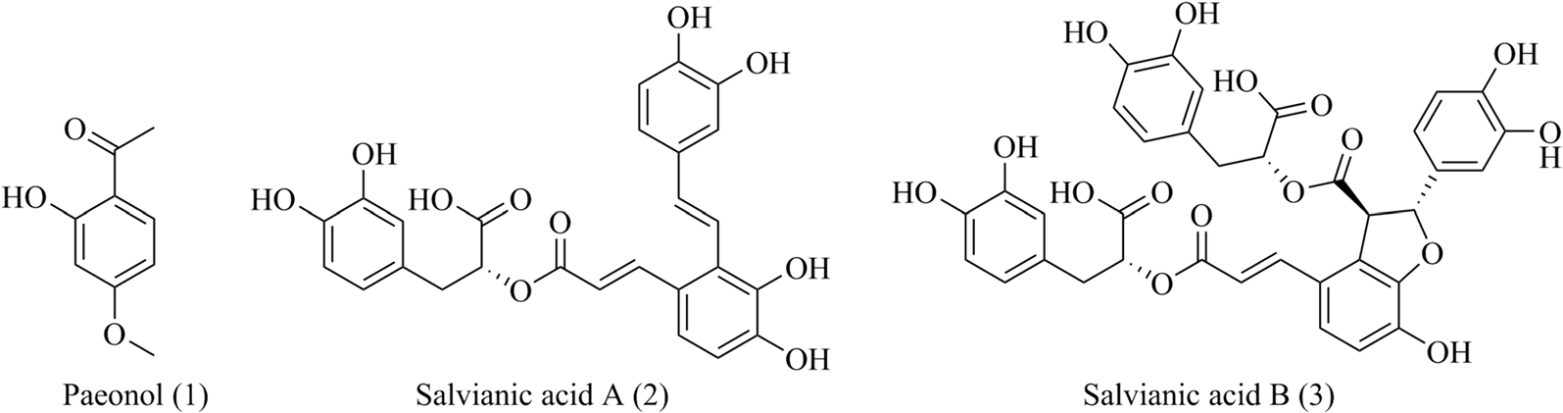
Chemical structures of polyphenols, including paeonol, salvianic acid A and salvianolic acid B

Paeonol (**1**, Fig. 3) is an active polyphenol from the root bark of *Paeonia suffruticosa* Andr*.* In previous study, paeonol exhibited protective effect on acute myocardial infarction rats by inhibiting LTCCs currents in rat ventricular myocytes [47, 48]. In addition, paeonol induced non-endothelium dependent-vasodilatation in rat mesenteric artery by inhibiting VGCCs via inducing extracellular Ca^2+^ influx [49]. Therefore, the mechanism of paeonol in reducing myocardial infarction and protecting myocardial cells from hypoxia injury may be related to inhibition of LTCCs. In another study, paeonol alleviated primary dysmenorrhea by inhibiting Ca^2+^ influx and uterine contraction via cannabinoid receptor 2 (CB2R) in uterine smooth muscle cells through MAPK/ERK pathway. As a result, paeonol exhibited the similar effect as positive control, nifedipine, in suppressing uterine contraction in vitro [50].

#### Salvianic acid A and salvianolic acid B

Salvianic acid A and salvianolic acid B (**2**–**3**, Fig. 3) are the main water-soluble constituents from *Salviae Milthiorrhizae* Bge., which have been used to treat cardiovascular diseases such as stroke, myocardial infarction and hypertension [51]. Salvianolic acid A and salvianolic acid B exerted cardioprotective effects by reducing L-type Ca^2+^ channel currents (I_Ca,L_), upshifting the current–voltage, leftshifting the curves of activation and inactivation, and inhibiting the amplitude of the cell shortening [52, 53]. Moreover, salvianolic acid B showed vasorelaxant effects on isolated coronary artery rings precontracted with 5-hydroxytryptamine by inhibiting Ca^2+^ influx in the vascular smooth muscle cells [54].

### Flavonoids

#### Luteolin

**Fig. 4:**
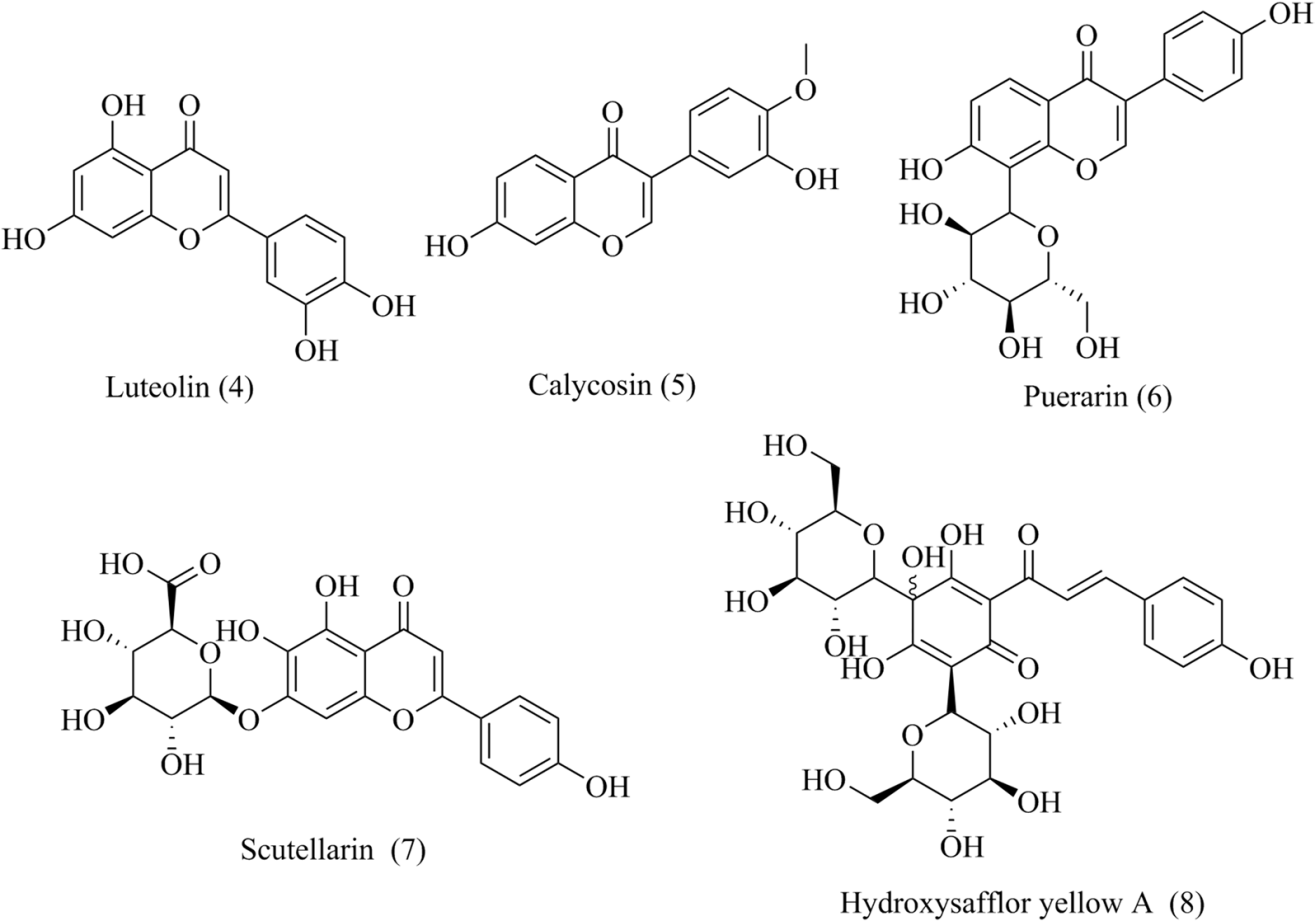
Chemical structures of flavonoids, including luteolin, calycosin, puerarin, scutellarin and hydroxysafflor yellow A

Luteolin (**4**, Fig. 4) is a natural flavonoid isolated from many traditional medicines and has various pharmacological activities in osteoporosis, allergy, diabetes, tumors and liver toxicity [55]. The large amount of oxygen free radicals and calcium overload in myocardial cells are the main causes of heart injury. Furthermore, calcium influx through LTCCs during ischemia and hypoxia lead to further overload of calcium storage. Luteolin showed protective effect on heart from long-term preservation damage, such as structural damage, heart dysfunction and increased apoptosis by inhibiting hypoxia-dependent L-type calcium channels, which suggested the usage of luteolin as heart preservation solutions, especially in long-term storage [56].

#### Calycosin

Calycosin (**5**, Fig. 4) is an isoflavonoid isolated from Radix Astragali (*Astragalus membranaceus* (Fisch.) Bunge.), and showed the vasorelaxant effect on denuded and intact endothelium aortic rings [57]. Calycosin reduced aortic ring contractions induced by agonists KCl and PHE and its vasorelaxant action was different from that of dihydropyridines. The study revealed that calycosin was a noncompetitive Ca^2+^ channel blocker that its vasorelaxant effect was endothelium-independent and was unrelated to intracellular Ca^2+^ release [57].

#### Puerarin

Puerarin (**6**, Fig. 4) is an isoflavone *C*-glucoside isolated from *Pueraria lobata* (Willd.). The increased intraocular iron stimulates ROS generation lead to retinal injury. ROS showed the biphasic effect on the Ca^2+^ transport in cells: on one side, ROS reduced the Ca^2+^-ATPase of sarcoplasmic reticulum (SR), which can diminish the Ca^2+^ level in the cell; and on the other side, ROS deactivated the plasma membrane Ca^2+^-ATPase, which increase the Ca^2+^-loading and ultimately leads to apoptosis [58]. Puerarin was reported the protection effect against retinal injury caused by iron overload though reducing the Cav1.2 expression in retinal tissue [59]. Puerarin inhibited the level of Cav1.2 expression in ARPE-19 cells, indicating that puerarin attenuated the iron deposition by regulating the iron-handling proteins [59]. The above results suggested that LTCC might be a potential target for puerarin on iron-mediated retinal injury. However, the specific target of puerarin inhibiting Cav1.2 needs further study.

#### Scutellarin

Scutellarin (**7**, Fig. 4) is a flavonoid glycoside from *Erigeron breviscapus* Hand Mazz. It had been reported that LTCC was activated by CaMKII, and CAMKII-mediated changes in calcium current may be associated with cardiovascular disease [60]. The Ca^2+^-calcineurin and CaMKII were two important effector of Ca^2+^-medicated cardiac hypertrophy [61]. In this study, scutellarin suppressed the cardiac hypertrophy exposed to phenylephrine (PHE) by inhibiting the Ca^2+^-mediated calcineurin and CaMKII pathways [61]. Accordingly, scutellarin may be used as candidate against cardiac hypertrophy in future.

### Hydroxysafflor yellow A

Hydroxysafflor yellow A (**8**, Fig. 4) is a water soluble constituent from *Carthamus tinctorius* L. and exerts various effects on cardiovascular diseases [62]. Hydroxysafflor yellow A showed the cardioprotective effect on HR-induced myocardial injury in neonatal rat primary cardiomyocytes (NPCMs) and human-induced pluripotent stem cell-derived cardiomyocytes (hiPSC-CMs). Further study showed that hydroxysafflor yellow A attenuated the expression of *α*1C and α2*δ* subunits of LTCC in vivo and in vitro*.* Bay-K8644, an LTCC agonist, was used to stimulate the LTCC excessive in study. As a result, Hydroxysafflor yellow A inhibited the electrical signal disturbances and the higher calcium currents caused by the excessive activation of LTCC in hiPSC-CMs, suggesting that hydroxysafflor yellow A treated MI/RI via regulating LTCC to inhibit Ca^2+^ overload and apoptosis [63].

### Terpenoids

#### Safranal

Safranal (**9**, Fig. 4), an active monoterpene derived from *Crocus sativus* L. (saffron). Safranal protected MI injury induced by Isoproterenol (ISO) in rats via regulating Ca^2+^ homeostasis, inhibiting oxidative stress and reducing cardiac systolic dysfunction [64]. Specifically, safranal decreased the cell contraction, Ca^2+^ transient and I_Ca-L_ in myocardial cells [64].

#### Paeoniflorin

Radix Paeoniae Alba, the root of *Paeonia lactiflora* Pall, has the effect of relieving depression and regulating menstruation in Chinese medicine. Paeoniflorin (**10**, Fig. 4) is the main bioactive terpene glycoside of paeony extract and has anti-depressive and neuroprotective effects.

It was confirmed that paeony extract and Shuyu capsule improved the depressive hehaviour, such as body weight, open-field test scores, and sucrose preference in premenstrual syndrome (PMS) rats by regulating Cav1.2 mediated CaM/CaMKII signalling. Further results revealed that paeoniflorin inhibited intracellular Ca^2+^ overloading induced by K^+^ and inhibited Cav1.2 current density in a dosage-dependent manner [65]. Thus, paeoniflorin played an antidepressant role by mediating LTCCs.

#### Ginsenosides

**Fig. 5:**
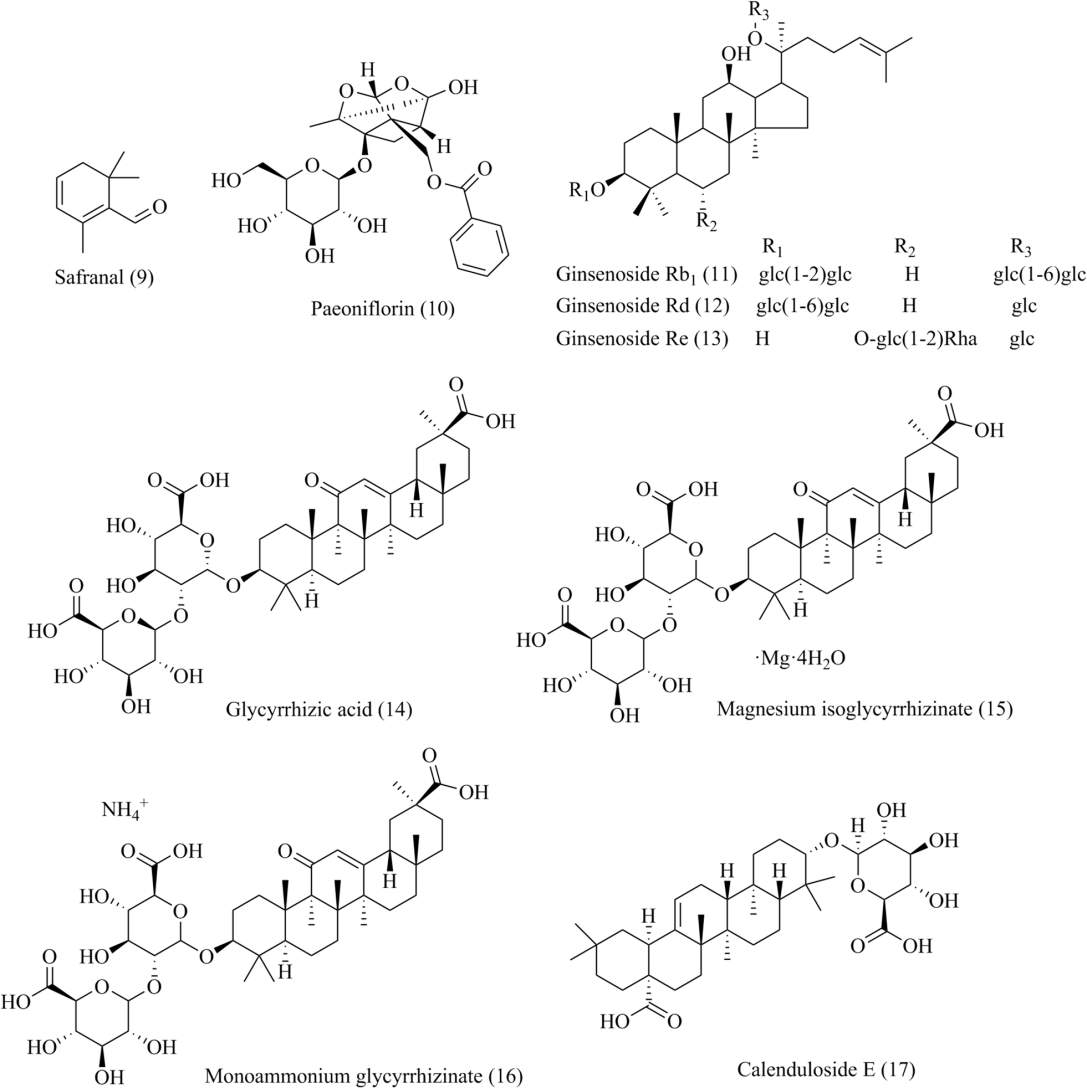
Chemical structures of terpenoids, including safranal, paeoniflorin, ginsenosides, glycyrrhizic acid and calenduloside E

Ginsenosides are the major bioactive ingredients from *Panax ginseng*. The total ginsenosides (TG) displayed cardioprotective effects on ISO-induced MI rats by inhibiting of I_Ca-L_, myocytes shortening and Ca^2+^ transient [66]. Ginsenoside Rb_1_ and ginsenoside Rd (**11**–**12**, Fig. 5) have been reported the anti-MI effect in rat ventricular myocytes by inhibiting L-type Ca^2+^ current in a dosage-dependent manner [67–69]. Ginsenoside Rb_1_ and Re (**13**, Fig. 5) inhibited the mRNA expression of Cav1.2 on rat cardiomyocyte injury induced by aconitine alkaloids [70]. Further study revealed that the inhibition of I_Ca,L_ induced by ginsenoside Rd was abolished by pertussis toxin, a Gi protein inhibitor, suggesting that Gi protein was the potential target of Rd for treating MI/RI in rat [69].

#### Glycyrrhizic acid and its derivatives

Glycyrrhizic acid (**14**, Fig. 5) is a bioactive component isolated from *Glycyrrhiza uralensis* Fisch with wide range of pharmacological effects such as antiviral, anti-tumor, anti-inflammatory, bactericidal, and anti-MI [71]. The neuroprotective mechanisms of glycyrrhizic acid was verified to be related with modulating multiple anti-apoptotic and pro-apoptotic factors and inhibiting intracellular Ca^2+^ overload [72]. Glycyrrhizic acid exhibited the cardioprotective effects by inhibiting L-type Ca^2+^ channels and reducing the Ca^2+^ transient in ISO-induced myocardial ischemia injury rats [73]. Specifically, Glycyrrhizic acid decreased the elevation of ST segment, reduced the heart rate, increased the QT-interval shortening induced by ISO, and amended the heart morphology. Furthermore, Glycyrrhizic acid blocked L-type Ca^2+^ channels in a dose-dependent manner and reduced the Ca^2+^ transient in the rats ventricular myocardial cells [73].

The derivatives of glycyrrhizic acid also have similar biological functions. It has been reported that the calcium antagonists can suppress I_Kr_ in ventricular myocardial cell that causes long QT syndrome (LQTS), which was a serious disease with a high risk of developing cardiac arrhythmias [74]. An isomerized derivatives of glycyrrhizic acid, Magnesium isoglycyrrhizinate (**15**, Fig. 5), exerted cardiovascular protective effect by restraining I_Ca-L_ and inhibiting Ca^2+^ transient and decreasing myocardial contractility [75]. In addition, Magnesium isoglycyrrhizinate showed no effect on the expression of I_Kr_ in HEK293 cells, indicating that the usage of Magnesium isoglycyrrhizinate may not bring out drug-induced LQTS [75]. Monoammonium glycyrrhizinate (**16**, Fig. 5), an ammonium salt hydrate of glycyrrhizic acid, often clinically applied in treating viral hepatitis. Monoammonium glycyrrhizinate protected cardiomyocytes in ISO-induced MI model by inhibiting LTCCs and reducing oxidative stress, with the similar mechanism as glycyrrhizic acid [76]. Above results suggested derivatives of glycyrrhizic acid may be a promising drug for treating cardiovascular disease.

#### Calenduloside E

*Aralia elata* (Miq.) Seem is a traditional Chinese medicinal plant used for treating arrhythmia, diabetes and coronary heart disease. Calenduloside E (**17**, Fig. 5), a pentacyclic triterpenoid saponin from *Aralia elata* (Miq.) Seem., has the anti-MI, anti-hypoxia and anti-endothelial injury activity [77]. Calenduloside E was verified the protection effect by suppressing calcium overload though restoring the expression of calcium transporters, such as SERCA, *α*_1_C(Cav1.2), RyR2 and NCX, and regulating the calcium transients in MI/RI rats [77]. Further research showed that Calenduloside E enhanced the interaction between LTCCs and Bcl2-associated athanogene 3 (BAG3), specifically by inhibiting the *α*_1_C (Cav1.2) and *α*_2_*δ* subunits of LTCCs, restoring the interaction between BAG3 and *α*_1_C to alleviated MI/R injury [77]. In addition, the mechanism of calenduloside E has been found to be similar to that of nisoldipine, a dihydropyridine calcium channel blocker, suggesting that calenduloside E has the potential to be developed as an LTCCs antagonist.

### Alkaloids

#### Sinomenine

**Fig. 6:**
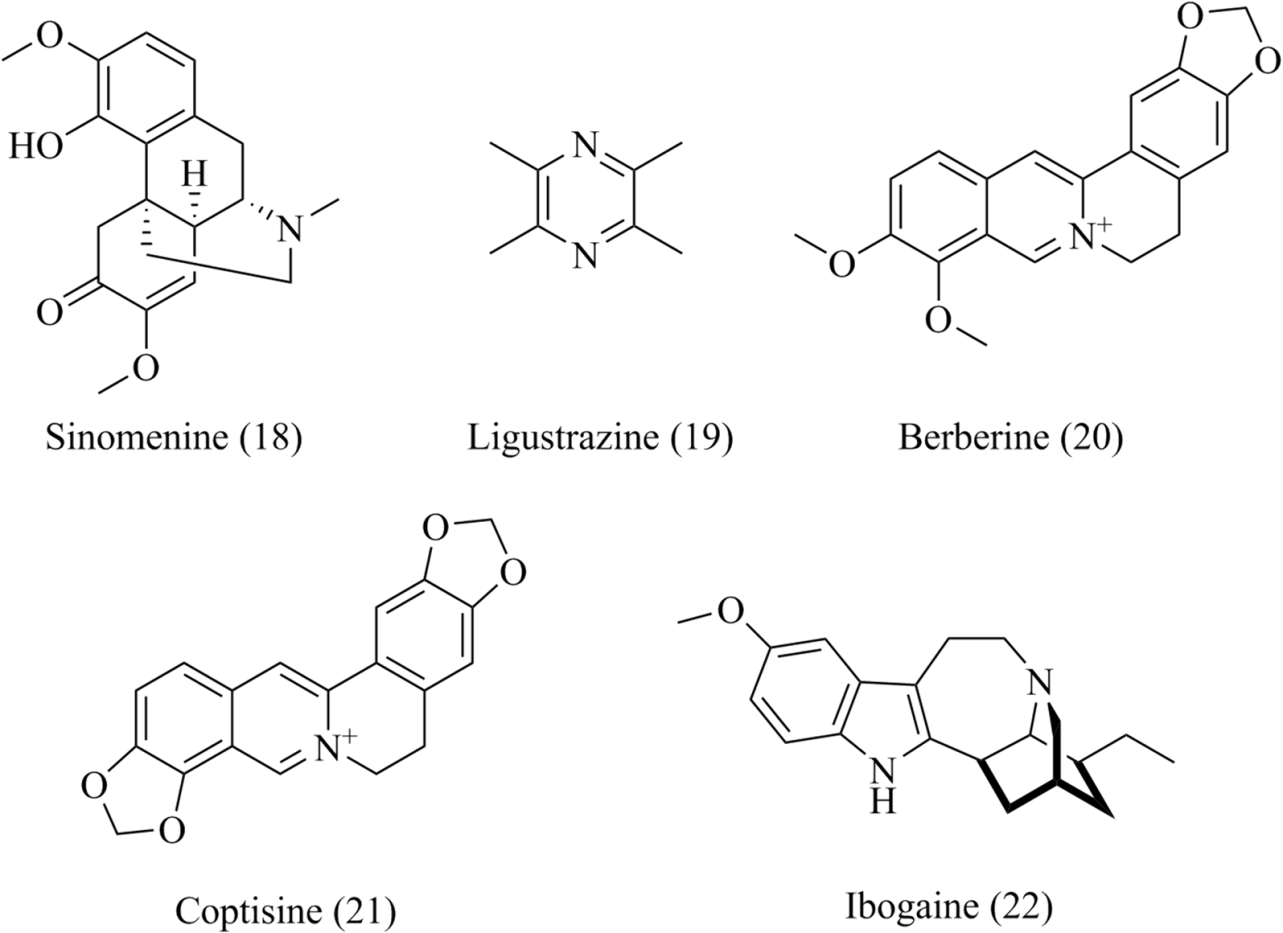
Chemical structures of alkaloids, including sinomenine, ligustrazine, berberine and coptisine

Sinomenine (**18**, Fig. 6), a major bioactive alkaloid from *Sinomenium acutum*, has protective effects on cardio-cerebrovascular system [78]. Sinomenine protected against the oxygen–glucose deprivation-reperfusion induced neurotoxicity in PC12 cell, and improved functional recovery in cerebral ischaemia rats [79]. Specifically, sinomenine inhibited L-type calcium currents, decreased [Ca^2+^]i induced by acidification, and reduced ASIC1a currents, which directly induced Ca^2+^ entry in rat cultured cortical neurons [79]. The sinomenine is expected to be applied in the prevention and treatment of stroke.

#### Ligustrazine

Ligustrazine (**19**, Fig. 6) is an alkaloid purified from *Ligusticum wallichil* and has been reported to be a calcium antagonist in treating cardiovascular and cerebrovascular diseases [80]. Ligustrazine protected cardiomyocytes against Ischemic heart disease (IHD) by inhibiting I_Ca,L_, reducing intracellular Ca^2+^ overload, and surpressing calcium transient in rabbit ventricular myocytes [81]. Ligustrazine alleviated musculoskeletal disorders (MSD) in rats caused by static posture load via enhancing the activity of Ca^2+^-ATPase, inhibiting expression of LTCC Cav1.3 and maintaining the homeostasis of Ca^2+^ in soleus muscle cells [82].

In addition, the neuroprotective effects of ligustrazine have also been reported [83, 84]. Ligustrazine showed protective effects on hippocampal neuron cells mainly by inhibiting I_Ca,L_ and reducing intracellular calcium concentration [83]. Moreover, the protection of ligustrazine on SH-SY5Y human neuroblastoma cells by inhibiting LTCC were reported [84].

#### Berberine and coptisine

Berberine and coptisine (**20**–**21**, Fig. 6) are active alkaloids widely existing in *Coptis* species, which have anti-tumor, anti-microbial and cardio-cerebrovascular protection effects [85]. Berberine treatment inhibited LTCCs by decreasing the expression of *α*_1_C subunit and the intracellular Ca^2+^ level in smooth muscle cells of streptozotocin-induced diabetes rats [86]. Berberine enhanced the neuroprotective effect of verapamil in sporadic dementia of Alzheimer’s type rats induced by intracerebroventricular streptozocin by inhibiting of P-gp efflux and regulating calcium homeostasis [87]. Berberine exerted the positive inotropic effect on left ventricular myocytes of rat heart by enhancing the Ca^2+^ influx [88]. In addition, bromibenzyltetrahydroberberine (CPU86035), a tetrahydroberberine derivative, strongly inhibited LTCCs in single ventricular myocyte of guinea pig, which can be used in the treatment of myocardial infarction [89]. Therefore, the effects of berberine and its derivatives may provide therapeutic strategies for calcium channel diseases.

Coptisine relaxed abnormal contracted mouse airway smooth muscle (ASM) by eliminating LTCCs and and regulating intracellular Ca^2+^ concentration, and exhibited the similar calcium antagonism as nifedipine [90].

#### Ibogaine

Ibogaine (**22**, Fig. 6), an indole alkaloid isolated from the root bark of the African shrub *Tabernanthe iboga* with a long history usage as medicinal agent to treat drug abuse in local aera [91]. The study revealed that its anti-addictive effect of ibogaine was associated with the inhibition of Cav 1.2 channel in guinea pig cardiomyocytes [92].

### Steroids

#### Cinobufagin and bufalin

**Fig. 7:**
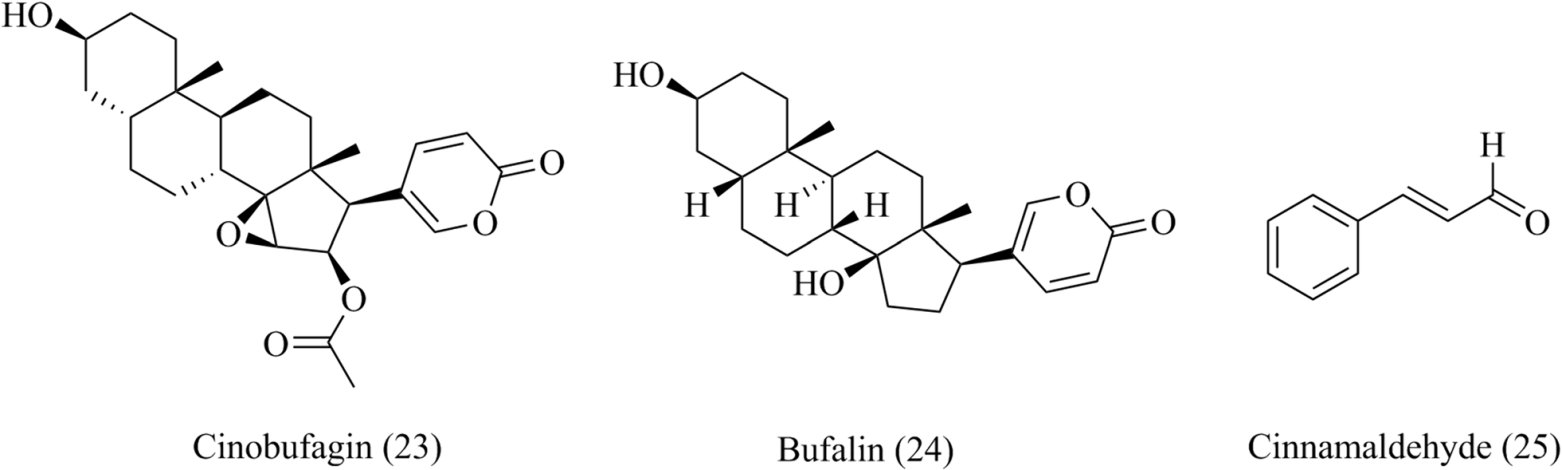
Chemical structures of steroids and phenylpropanoids, including cinobufagin, bufalin and cinnamaldehyde

Chan Su, a Chinese medicine made from the dried white secretions of Chinese toads (*Bufo melanostictus* Schneider or *Bufo bufo gargarizans* Cantor) [93]. Cinobufagin and bufalin (**23**–**24**, Fig. 7), two natural bufaldienolides from Chan Su, have been reported the cardioprotective effect by inhibiting LTCCs. Cinobufagin induced certain electrophysiological changes in the properties of I_Ca,L_ in a concentration–dependent manner [93]. Bufalin inhibited the I_Ca,L_, and contractility in rat ventricular myocytes induced by Bay K8644, an LTCCs agonist [94].

### Phenylpropanoids

#### Cinnamaldehyde

Cinnamaldehyde (**25**, Fig. 7), an active natural ingredient from the *Cinnamomum tamala* (Bauch.-Ham.), has been used in treating cardiovascular diseases [95]. Cinnamaldehyde exhibited complex effects on rat aortic rings and isolated mouse hearts by activating the chemosensory cation channel TRPA1 as well as inhibiting LTCCs. The inhibitory action of cinnamaldehyde on I_Ca,L_ in both ventricular cardiomyocytes (VCM) and vascular smooth muscle cells(VSMC) was obtained and these effects were similar to those of classic LTCCs blocker verapamil [95].

### Others

#### Salidroside

**Fig. 8:**
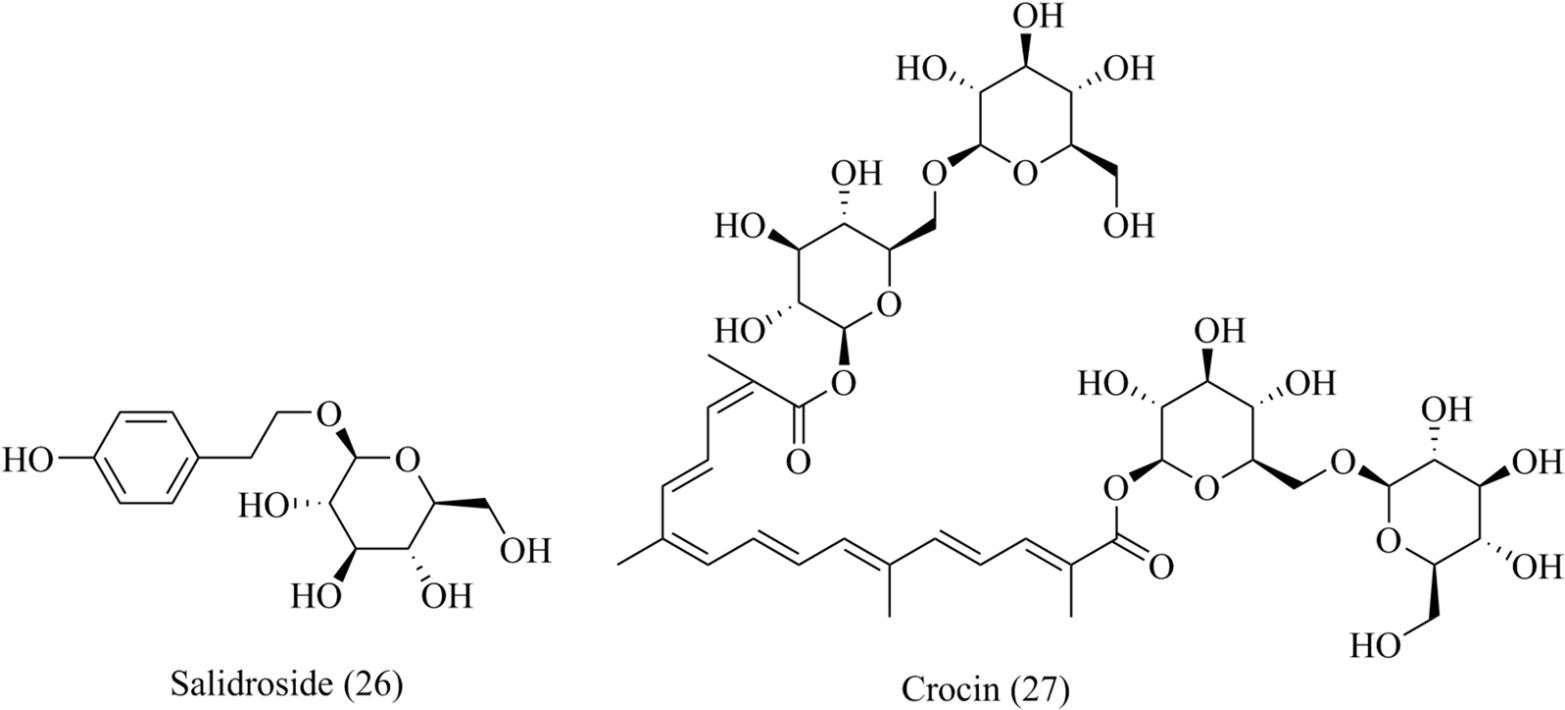
Chemical structures of others, including salidroside and crocin

Salidroside (**26**, Fig. 8) is a phenylethanoid derivative in *Rhodiola rosea* L. and has obvious hypoglycemic effect in diabetes. Recently, studies have explored the antihypertensive mechanism of salidroside in diabetic vascular complications. Salidroside dilated the cerebral arteries of diabetic rats, but could not recover to the normal level, and had no diastolic effect on the cerebral arteries of normal rats. In this process, calcium current density, the protein and mRNA expressions of *α*_1_C subunit at diabetic rats were inhibited by salidroside [96]. Salidroside also showed the protection hippocampal neurons against hypoxic-induced injury based on inhibiting LTCCs and reducing the mRNA expression levels of Cav1.3 and NMDAR1 to alleviate the intracellular calcium overload [97].

#### Crocin

Crocin (**27**, Fig. 8), a major bioactive substance from *Crocus sativus* L., have various effects on cardio-cerebrovascular system, including anti-oxidation, anti-MI and MI/RI [98]. In terms of anti-MI, crocin exerted negative inotropic effects on myocardium, reduced oxygen consumption and protected myocardium cells by inhibiting I_Ca,L_ and reducing Ca^2+^ influx [99].

### LTCCs activators from natural products

**Fig. 9:**
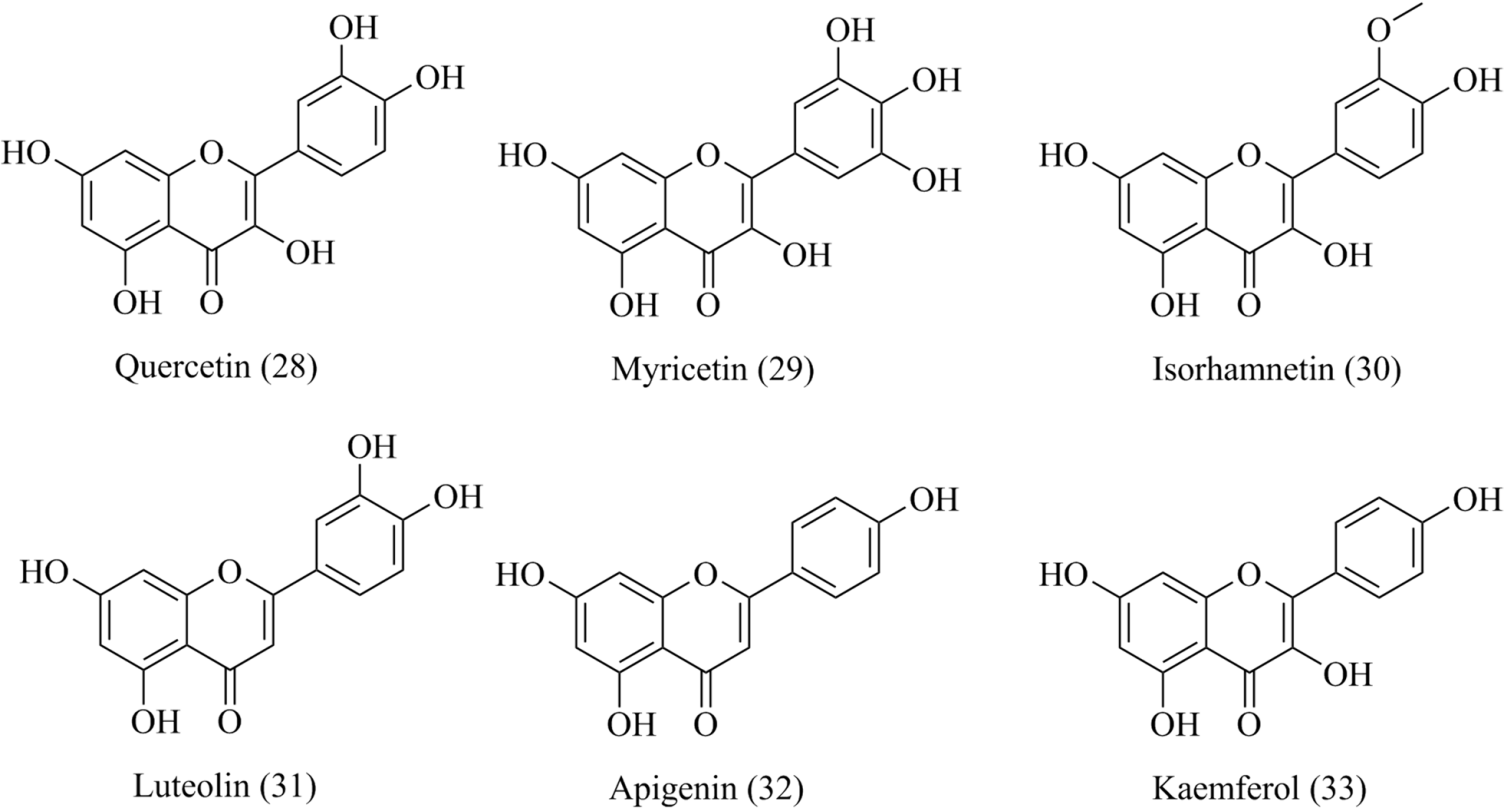
Chemical structures of quercetin, myricetin, isorhamnetin, luteolin, apigenin and kaempferol

There are few studies on LTCCs activators in TCM. Studies have shown that quercetin (**28**, Fig. 9, 10 μM) activated Cav1.2 channel current (I_Ca1.2_), negative shifted the steady-state inactivation curve and slowed recovery rate from inactivation in rat tail artery [100]. However, the electrophysiological features of quercetin on I_Ca(L)_ were different from Bay K 8644, a known Ca^2+^ channel agonist. The in-depth research showed that the ineffective concentrations of quercetin (0.1 and 0.3 μM) inhibited the max response induced by Bay K 8644, indicating that low dosage of quercetin may restricted the LTCC reaction stimulated by Bay K 8644 [101]. Another study showed that quercetin induced insulin secretion by directly activating LTCCs in insulin-secreting cell line INS-1, which has potential for controlling type 2 diabetes [102]. Myricetin (**29**, Fig. 9), a analogue of quercetin, exerted the similar activatation on L-type Ca^2+^ channel with (*S*)-(-) Bay K 8644, by slowing down the activation kinetics and stabilizeing the channel in its inactivated state [103, 104].

The regulatory ability of flavonoid constituents on Cav1.2 channels were summarized [105]. Twenty-four flavonoids were conducted to measure their effects on I_Ca1.2_ in rat tail artery myocytes with patch-clamp Method. As a result, the effect of flavonoids on calcium channels is voltage dependent, six compounds including quercetin (**28**), myricetin (**29**), isorhamnetin (**30**), luteolin (**31**), apigenin (**32**) and kaempferol (**33**) enhanced the I_Ca1.2_ with the EC_50_ of ranging between 2.9 and 16.0 mM (Fig. 9). The affinity of quercetin to Cav1.2 was 3 times that of myricetin, and the effect of quercetin was significantly better than that of myricetin. The hydroxyl position and the double bond between C2 and C3 in skeleton of flavonoids were the important determinants for predicting the activity of flavonoids on calcium channels by molecular modelling method [105].

## Discussion

**Fig. 10:**
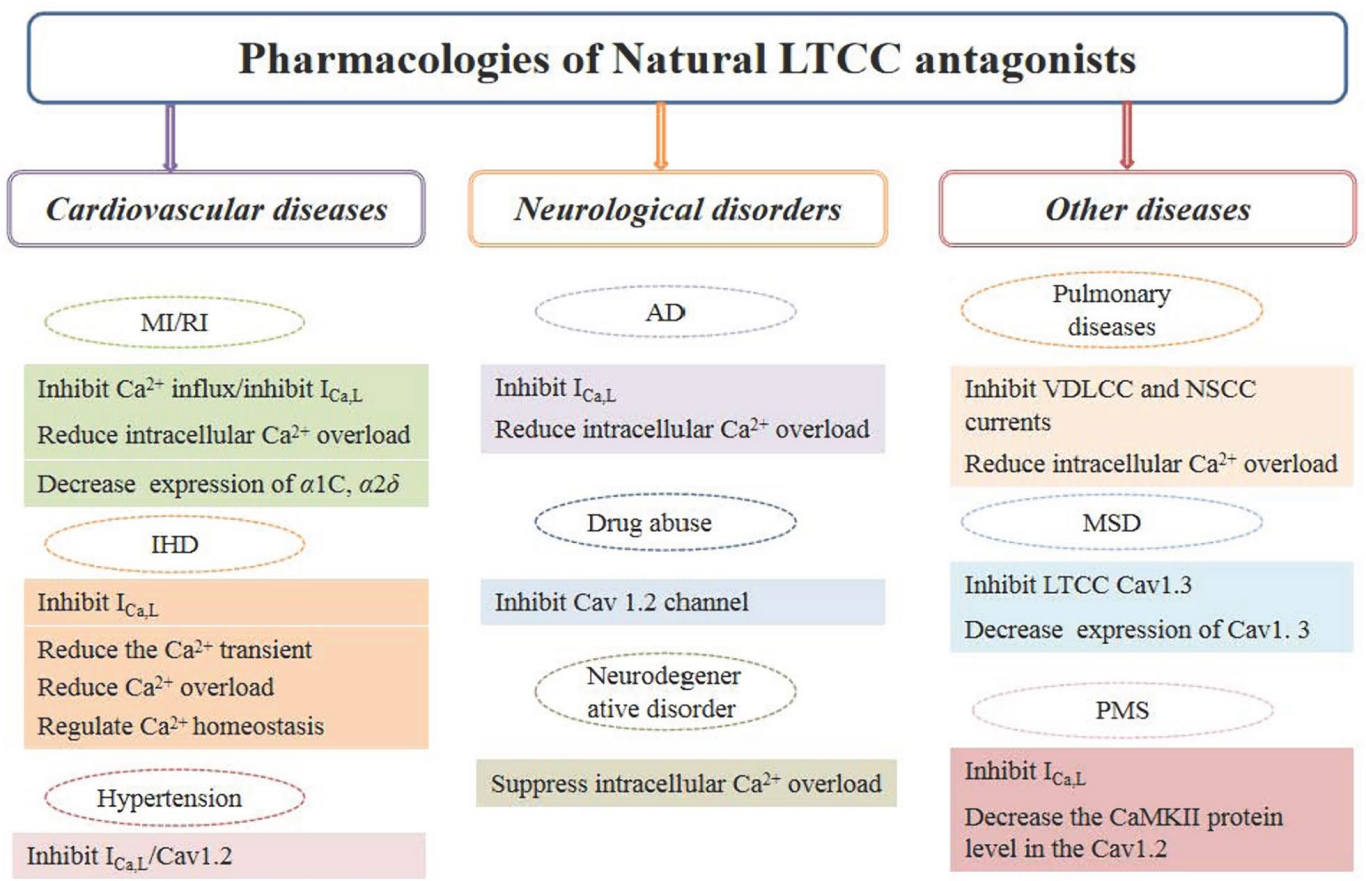
Pharmacological summary of Natural LTCC antagonists

LTCCs is an important voltage-gated channel that are responsible for regulating intracellular calcium balance and participating in a variety of human diseases, which has been considered as the potential therapeutic target. Abnormal LTCCs expression is closely related to the progression of cardiovascular, neurological and psychological diseases. Correcting calcium homeostasis disorders may be successful therapeutic strategies in the treatment of above diseases or delay the progression of diseases [106]. Many non-natural LTCCs antagonists have been used in clinical practice for decades, such as nimodipine, diltiazem and verapamil [11]. Multiple types of natural LTCCs antagonists from TCM, including polyphenols, flavonoids, terpenoids, alkaloids, steroids and phenylpropanoids were summarized in this review (Fig. 10). Most natural LTCCs antagonists mentioned in the article were isolated from herbs (92.6%), but part of them, such as Cinobufagin and bufalin, were obtained from animal (7.4%) [93, 94]. Animal medicine was one of the main sources of TCM, that has received enough attention in TCM research. However, it is necessary to pay attention to the sustainable utilization of resources and the protection of ecological environment during the exploration of new animal medicine resources [107]. Actually, these natural LTCCs antagonists have been still in laboratory stage and not used in clinic so far.

Research revealed that non-selective calcium channel antagonists were dose-limited clinically by vascular effects and were prone to cause adverse reactions such as peripheral oedema, headache and dizziness [108]. Currently, the selective Cav1.3 blockers had significant therapeutic effects but without those vascular side effects of non-selective LTCCs blockers [43]. Therefore, specific LTCCs antagonists need to be developed in future. Furthermore, the LTCCs were new promising targets for many diseases, such as drug-addiction [33, 34], depressive disorder [109], age-related macular degeneration-retinal pigment epithelium (AMD-RPE) [110], intrauterine growth restriction [111], local infiltration analgesia [112], myalgia [113], which expanded the scope of application. In addition, calcium channel blocker (CCB) have a short plasma half-life especially in rodents and show high first-pass metabolism upon oral application [114]. The dosage of CCB should be tightly noticed as the CCBs lose specificity for their specific receptors and can show all the manifestations of toxicity such as bradycardia, peripheral vasodilation, and hypotension in high concentrations [115].

## Conclusions

LTCCs is a promising target to develop as its involvement in various heritable complex diseases..Nowadays, many natural products from TCM have been reported the inhibitary activity on LTCCs, but their molecular characteristics, intricate mechanisms, and shortage of clinical evidence limits their development and usage. Thus, the specific LTCCs antagonists with distinct clinically evidence should be discovered in future.

## Data Availability

Data sharing is not applicable to this article.

## Abbreviations

AD: Alzheimer’s disease
AMD-RPE: Age-related macular degeneration-retinal pigment epithelium
APAs: Aldosterone-producing adenomas
ASM: Airway smooth muscle
BAG3: Bcl2-associated athanogene 3
BD: Bipolar disorder
BrS: Brugada syndrome
CaMKII: Calmodulin-dependent protein kinase II
CB2R: Cannabinoid receptor 2
CCB: Calcium channel blocker
CHI: Congenital hearing impairment
CSNB2: Congenital stationary night blindness type 2
ECC: Excitation–contraction coupling
hiPSC-CMs: Human-induced pluripotent stem cell-derived cardiomyocytes
HPP-1: Hypokalemic periodic paralysis type 1
IHD: Ischemic heart disease
ISO: Isoproterenol
LQTS: Long QT syndrome
LTCCs: L-type calcium channels
I_Ca,L_: L type Ca^2+^ channel currents
MI: Myocardial ischemia
MI/RI: Myocardial ischemia/reperfusion injury
MSD: Musculoskeletal disorder
NCX: Sodium/calcium exchanger
NPCMs: Neonatal rat primary cardiomyocytes
PD: Parkinson’s disease
PHE: Phenylephrine
PMS: Premenstrual syndrome
RyR1: Type 1 ryanodine receptor
SD: Sprague–Dawley
SR: Sarcoplasmic reticulum
TCM: Traditional Chinese medicine
TS: Timothy syndrome
TSC: Tuberous sclerosis complex
VCM: Ventricular cardiomyocytes
VGCCs: Voltage-gated calcium channels
VSMC: Mesenteric artery smooth muscle cells
ZG: Zona glomerulosa
